# Development of a bivalent protein-based vaccine candidate against invasive pneumococcal diseases based on novel pneumococcal surface protein A in combination with pneumococcal histidine triad protein D

**DOI:** 10.3389/fimmu.2023.1187773

**Published:** 2023-08-23

**Authors:** Elnaz Afshari, Reza Ahangari Cohan, Mohammad Sadegh Shams Nosrati, Seyed Fazlollah Mousavi

**Affiliations:** ^1^ Department of Biology, Science and Research Branch, Islamic Azad University, Tehran, Iran; ^2^ Department of Microbiology, Pasteur Institute of Iran, Tehran, Iran; ^3^ Department of Nanobiotechnology, New Technologies Research Group, Pasteur Institute of Iran, Tehran, Iran; ^4^ Department of Internal Medicine and Medical Specialties (DIMI), University of Genova (UniGe), Genoa, Italy

**Keywords:** PspA, PhtD, serotype-independent, bivalent pneumococcal vaccine, opsonophagocytosis, serum bactericidal assay

## Abstract

Extensive efforts have been made toward improving effective strategies for pneumococcal vaccination, focusing on evaluating the potential of multivalent protein-based vaccines and overcoming the limitations of pneumococcal polysaccharide-based vaccines. In this study, we investigated the protective potential of mice co-immunization with the pneumococcal PhtD and novel rPspA proteins against pneumococcal sepsis infection. The formulations of each antigen alone or in combination were administered intraperitoneally with alum adjuvant into BALB/c mice three times at 14-day intervals. The production of antigen-specific IgG, IgG1 and IgG2a subclasses, and IL-4 and IFN-γ cytokines, were analyzed. Two *in vitro* complement- and opsonophagocytic-mediated killing activities of raised antibodies on day 42 were also assessed. Finally, the protection against an intraperitoneal challenge with 10^6^ CFU/mouse of multi-drug resistance of *Streptococcus pneumoniae* ATCC49619 was investigated. Our findings showed a significant increase in the anti-PhtD and anti-rPspA sera IgG levels in the immunized group with the PhtD+rPspA formulation compared to each alone. Moreover, the results demonstrated a synergistic effect with a 6.7- and 1.3- fold increase in anti-PhtD and anti-rPspA IgG1, as well as a 5.59- and 1.08- fold increase in anti-PhtD and anti-rPspA IgG2a, respectively. Co-administration of rPspA+PhtD elicited a mixture of Th-2 and Th-1 immune responses, more towards Th-2. In addition, the highest complement-mediated killing activity was observed in the sera of the immunized group with PhtD+rPspA at 1/16 dilution, and the opsonophagocytic activity was increased from 74% to 86.3%. Finally, the survival rates showed that mice receiving the rPspA+PhtD formulation survived significantly longer (100%) than those receiving protein alone or PBS and exhibited the strongest clearance with a 2 log_10_ decrease in bacterial load in the blood 24h after challenge compared to the control group. In conclusion, the rPspA+PhtD formulation can be considered a promising bivalent serotype-independent vaccine candidate for protection against invasive pneumococcal infection in the future.

## Graphical Abstract

Schematic overview of bivalent pneumococcal protein vaccine development comprising the novel rPspA and PhtD, and investigation of their protective immune responses against invasive pneumococcal infection.

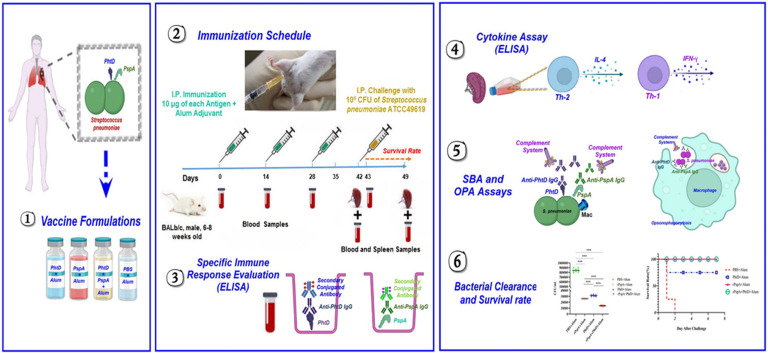

## 1 Introduction

*Streptococcus pneumoniae* (pneumococcus) is an opportunistic pathogen. According to the latest reports of the Centers for Disease Control and Prevention (CDC), pneumococci are classified into more than 100 distinct serotypes based on their capsular polysaccharide so far (https://www.cdc.gov/pneumococcal/laboratorians.html). Despite pneumococcus being transferred asymptomatically within the nasopharynx, it is the principal cause of a wide range of non-invasive diseases (NID) (e.g., pneumonia, sinusitis, otitis media, and conjunctivitis) as well as life-threatening invasive pneumococcal diseases (IPD) (e.g., bacteremia, meningitis, invasive pneumonia, and sepsis) (1–3). According to the assertions of the World Health Organization (WHO), pneumococcal infections with high morbidity and mortality worldwide are responsible for the annual death of 1.6 million people, of which 1 million deaths occur in children under the age of 5 (3–5).

Currently, the attractive alternatives to pneumococcal conjugate vaccines for preventing IPD are multivalent protein-based vaccine candidates that comprise combined formulations of conserved virulence factors with different functions in pneumococcal pathogenesis (1). It has been reported that these alternative candidates have many advantages compared to pneumococcal polysaccharide-based vaccines. They are simpler to produce than PPV or PCV, cover all pneumococcal serotypes, and avoid the issue of serotype replacement. They also are more immunogenic than polysaccharides in polysaccharide-based vaccines for children younger than 2 years old and elderly older than 65 years of age. In addition, the high cost of production of PCVs with the pipeline vaccine can be a limiting factor (6, 7). Besides, the opsonophagocytic antibodies raised against non-capsular protein antigens could offer serotype-independent protection against pneumococcal diseases (8). Furthermore, increasing evidence strongly indicates that a single protein of pneumococcus will not be adequate to produce protection against all strains of pneumococci. Therefore, the design of the multivalent protein-based formulations is under examination to assist in broad coverage and minimize the feasibility of immune escape (8, 9).

Various pneumococcal proteins have been studied as candidate antigens to design pneumococcal protein vaccine candidates (PPrVs) (9, 10). Pneumococcal histidine triad protein D (PhtD) is an extremely conserved surface-exposed protein, which is distinguished by the histidine triad (HxxHxH) motif presence (8, 10, 11). PhtD engages in zinc homeostasis and the attachment of pneumococcus to the host airway epithelium. This protein reduces complement deposition on the bacterial surface and prepares protection against pneumococcal colonization in the host nasopharynx and lung, as well as lethal infections in animal models (9, 10, 12). Recent studies have shown that PhtD in formulations alone or in combination with other pneumococcal proteins such as dPly or PcpA was the most protective, immunogenic, and safe in pre-clinical and clinical trials phase I/II studies (9, 13). Additionally, the effectiveness of the produced antibodies against PhtD was shown in a passive protection sepsis model (14). Pneumococcal surface protein A (PspA) is another attractive-conserved candidate for pneumococcal vaccines that has been widely studied (8). All pneumococcal strains express PspA on their surface to hinder the deposition of the complement on the surface and block lactoferrin’s bactericidal activity (15–18). PspA has three main domains, including a variable N-terminal domain with α-helices structure, a flexible cross-reactive region with features of repetitive motifs of proline residues as proline-rich domains (PRD), which a conserved non-proline block (NPB) region frequently interrupts, and finally a choline-binding region for anchoring the PspA to the pneumococcal surface (8, 19–21). Due to the accretion of mutations in the N-terminal region of PspA, a high degree of fickleness and variability in the amino acid sequence of PspA has been reported among different serotypes of pneumococci (8, 22, 23). Moreover, the 100 amino acids at the end of the PspA N-terminal region are recognized as a clade-defining region (CDR). Therefore, PspA has been divided into three families and six clades based on this CDR region, and they have various degrees of cross-reactivity (15, 19). Many PspA truncated domains or fusion proteins, as vaccine candidates, have been planned so far. It has been reported that elicited antibodies against the CDR and PRD regions can protect mice against the potentially fatal pneumococcal challenge (8, 24–28). In addition, immunization with PspA provided more protection than the licensed Prevnar conjugate vaccine in a murine model (29).

The experimental evidence firmly suggests that a single pneumococcal protein, particularly PspA from one clade or family, is not enough to promote protection against all strains of pneumococci (9, 30, 31). Besides, protective immunity against PspA is clade-dependent (23, 25, 32). Therefore, to overpower the PspAs clade-mediated immunity, in our previous study, we developed a promising novel PspA_1-5c+p_ candidate vaccine consisting of the truncated domain of the highest immunodominant coverage of B and T cell epitope from each clade focusing on cross-reactive regions of PspAs using immunoinformatics. We then investigated the cross-reactivity feature of the Anti-PspA_1-5c+p_ antibody against pneumococci expressing two PspA families 1 and 2 (23). According to the emphasis of recent studies on the use of multiple pneumococcal conserved proteins as multivalent vaccine candidates (8), in the current study, we determined the protective potential of immunization with our novel PspA_1-5c+p_ protein in combination with pneumococcal PhtD against pneumococcal sepsis infection in a mouse model and the effect of PhtD when combined with PspA_1-5c+p_ on specific anti-rPspA immune responses and vice versa. Based on our studies, the use of these two antigens as candidates for a bivalent pneumococcal vaccine has not been investigated so far.

## 2 Materials and methods

### 2.1 Bacterial strains and culture conditions

*E. coli BL21* (DE3) (Novagen) as an expression host and *Streptococcus pneumoniae* ATCC49619 (serotype 19F) (pneumococcus) were obtained from the bacterial collection of the Pasteur Institute of Iran. *E. coli BL21* was cultured on Luria-Bertani broth (LB) medium (Sigma Aldrich, USA) at 37°C. Pneumococcus was routinely grown on blood agar plates with 5% sheep blood or brain heart infusion broth (BHI) (Merck, Germany) at 37°C in 5% CO_2_.

### 2.2 Pneumococcal antigen preparation

In a previous study, we designed the new pneumococcal rPspA construct comprising the poly-epitope truncated domains of both PspA families 1 and 2 into the pET28a expression vector using restriction enzymes (*Nco*I and *Xho*I) and a 6-His tag at the C-terminus of the protein for easy purification (23). The National Center for Biotechnology Information (NCBI) server (https://www.ncbi.nlm.nih.gov) was used to retrieve the nucleotide sequence of the gene encoding pneumococcal *phtD* (Accession number: NC_003098). After codon optimization of the sequence, an *in-silico* cloning of the *phtD* gene into the pET28a expression vector was performed using the restriction enzymes (*BamH*I and *Hind*III) and the C-terminal 6-His tag by the SnapGene 6.0 software. Biomatik Corporation (Cambridge, Ontario, Canada) synthesized two constructs. The constructs were then transformed into *E. coli BL21* (DE3) competent cells via heat shock transformation (33). Positive clones were identified by restriction enzyme digestion and colony PCR with universal T7 primers (Pishgam Biotech Co., Iran) (T7 promoter: 5’-TAATACGACTCACTATAGGG, and T7 terminator: 5’-GCTAGTTATTGCTCAGCGG) and a PCR program with primer annealing at 55°C for the 30s and DNA extension at 72°C for 90s. For expression of recombinant rPspA and PhtD proteins, the transformed strains were inoculated in an LB broth medium containing 50 μg.ml^-1^ kanamycin and incubated at 37°C at 250 rpm to reach an optical density of 0.8 in 600 nm. Then, the expression was induced by adding 1mM Isopropyl-β-D-Thiogalactopyranoside (IPTG) (Thermo Fisher Scientific, USA) for 16 hours at 37°C. The results were evaluated by 12% SDS-PAGE and confirmed by transferring to PVDF Amersham™ Hybond®P membrane (GE Healthcare, Thermo Fisher Scientific, USA) for Western blotting using HRP conjugated anti-His-Tag antibody (Invitrogen Life Technologies, USA). The expressed recombinant proteins were then purified using a nickel affinity chromatography column (Ni-NTA chromatography) (Qiagen, Hilden, Germany) under modified hybrid conditions, followed by a buffer-exchange washing with decreasing urea concentrations (8, 6, 4, 2, and 0 M) (Sigma Aldrich, USA) and increasing imidazole concentrations (30, 40, 50, and 250mM) (Sigma Aldrich, USA). The purified proteins were freed of endotoxin using ϵ-poly-L-lysine-agarose (endotoxin removal spin column, Thermo Fisher Scientific, USA) and were dialyzed against PBS overnight at 4°C. Finally, we measured the concentration of the proteins with the Bradford assay (23, 34–36).

### 2.3 Vaccine formulations and systemic immunization

For this study, 6 to 8-week-old male BALB/c mice (n=28) were purchased from the Pasteur Institute of Iran (Karaj, Iran) and divided into four groups (7 mice/group). Each group was immunized intraperitoneally three times on days 0, 14, and 28 with different formulations of recombinant PhtD alone, rPspA alone, or PhtD+rPspA. Each mouse received 10 μg of each antigen alone (or in combination) in Alum adjuvant solution (Imject™ Alum Adjuvant-Thermo Fisher Scientific, USA) in a volume ratio (1:1 v/v) with the final volume of 200μl per mouse. The control group was injected with PBS and Alum. The retro-orbital bleeds were performed before each injection and 2 weeks after the last immunization, and pooled sera were collected from all blood samples in each group after 1 hour by centrifugation at 1200×g for 30 min and stored at -80°C until immunoassay (23, 34, 36–39).

### 2.4 Ethics approval

All animals were kept and handled in accordance with the ethical guidelines of the Institutional Animal Care and Use Committee at the Pasteur Institute of Iran (Approval ID: IR.PII.REC.1398.005).

### 2.5 Assessment of specific immune responses

We monitored the production of the specific IgG against rPspA and PhtD antigens in the serum of the immunized mice on days 14, 28, and 42 using indirect ELISA. Briefly, the 96-well ELISA plates (Nunc MaxiSorp, Thermo Fisher, USA) were coated with 100 μl of the recombinant PhtD alone or rPspA alone (1μg/well) in coating buffer (0.05 M carbonate-bicarbonate buffer, pH 9.6) overnight at 4°C. We used the capture antibody (purified anti-mouse IgG monoclonal antibody, Invitrogen, USA) in the coating buffer for the composition of the standard curve. The wells were then blocked with 5% bovine serum albumin (BSA; Sigma, USA) in PBS containing 0.05% Tween20 buffer (PBST buffer). Following washing three times with PBST, 100μl of 0.001 and 0.000001 diluted sera (for rPspA and PhtD antigens, respectively) in blocking buffer was added to the plates and incubated for 1 hour at 37°C. To measure the concentration of specific anti-PhtD and anti-rPspA IgGs, we also used serial dilutions of the mouse IgG isotype control (200-0 ng/ml) (Invitrogen, USA) to plot the standard curve. Afterward, the 1:10,000 dilution of HRP-conjugated anti-mouse IgG (Sigma, USA) was used as a detection antibody for 1 hour in a 37°C incubation. After washing, the plates were incubated with the tetramethylbenzidine (TMB) substrate (Thermo Fisher, USA) for 15 min in the dark and stopped with H_2_SO_4_ to measure antibody reactivity at 450 nm using an Epoch absorbance microplate reader (BioTek Company, USA) (23, 34, 40, 41). The well that contained antigens without serum was considered a negative control. All experiments were performed in triplicate and were given as mean ± S.D. One and two-way analysis of variance (ANOVA) followed by Tukey’s multiple comparison tests were performed to analyze the immune responses. P-values less than 0.05 were considered statistically significant.

### 2.6 Determination of IgG subclasses (IgG1 and IgG2a)

In order to evaluate the specific immune response towards humoral immunity or cellular immunity generated against each antigen in immunized mice groups with antigen alone or in combination, the subclasses of specific IgG (IgG1 and IgG2a) against PhtD and rPspA were measured using an indirect ELISA test. Similar to the indirect ELISA test described above, with the help of 1:10,000 dilution of HRP-conjugated anti-mouse IgG1 and IgG2a (Sigma, USA), the subclasses of sera IgG against each protein were checked in the serum of the immunized mice groups in day 42.

### 2.7 Cytokine assay

To investigate and confirm the T-helper-2 (Th-2) or T-helper-1 (Th-1) immune pathways created in the immunized groups with the purified antigens individually or in combination, the level of IL-4 and IFN-γ cytokines representing the Th-2 and Th-1 immune pathways, respectively, were assessed. For this purpose, the immunized mice from each group (n=3) were sacrificed 2 weeks after the last immunization, and their spleens were harvested and homogenized in cold RPMI 1640 (Gibco Co. USA) containing 10% FBS, 100 μg/ml Pen/Strep, and 2mM L-glutamine. The homogenized splenocytes were then centrifuged, and the red blood cells were eliminated from the single-cell suspension by treatment with RBC lysis buffer (eBioscience, Thermo Fisher, USA). Live splenocytes at a density of 1×10^6^ cells/well were cultured in 24-well microtiter plates (Greiner, Germany). Afterward, the spleen cells from each immunized group were stimulated with the same antigen that was immunized (rPspA (10 μg/ml) or PhtD (10 μg/ml), or rPspA plus PhtD (10 + 10 μg/ml)) and incubated for 72 hours at 37°C in 5% CO_2_. Finally, we assayed the concentrations of IFN-γ and IL-4 in the culture supernatants using the DuoSet ELISA kit (R&D Systems, USA) according to the manufacturer’s instructions (41, 42). The well that contains RPMI medium with non-stimulated splenocytes was considered the negative control. The cytokine assay for each group was performed in triplicate. Finally, the mean absorbance of each stimulated group was subtracted from the control group and was given as mean ± S.D.

### 2.8 Serum bactericidal assay

The serum bactericidal assay (SBA) as a functional antibody test was performed to evaluate whether the specific antibodies created in each group have an *in vitro* bactericidal activity using the complement system or not. In addition, the titer of required antibodies to kill bacteria following complement activation was measured (43). For this purpose, the 96-well polystyrene round bottom microwell plates (Thermo scientific NuncTM, USA) were loaded for 30 min at 37°C with 12.5μl of prepared pneumococcal suspension (*S. pneumoniae* ATCC 49619) at 10^6^ CFU/ml (based on the standard of 0.5 McFarland) and 12.5μl of dilutions of inactivated serum samples at 56°C for 30 min (1:2 to 1:64). Then, infant rabbit serum (4%) as a source of complement was added to each well. At two intervals (0 and 2 h), the content of each reaction well was serially diluted, and 10 μl from each well was inoculated into the blood agar plate. After 18-24 hours of incubation at 37°C in 5% CO_2_, the colony-forming unit of bacteria was measured. The titer of bactericidal antibodies is usually considered as the dilution of serum, which killed 50% of pneumococci compared to the control. The negative control was a well that contained bacteria and rabbit complement (23, 36, 40, 44, 45).

### 2.9 Opsonophagocytic killing activity

Another *in vitro* functional antibody test was performed to assess the opsonophagocytic killing (OPA) features of antibodies in the serum of the immunized BALB/C with rPspA alone, PhtD alone, or rPspA plus PhtD using phagocytic cells against the pneumococcal strain ATCC 49619. For this goal, the target pneumococcus was prepared at 10^6^ CFU/ml (based on the standard of 0.5 McFarland). To collect macrophage and neutrophil cells from the peritoneal cavity of the naïve mice, 10ml of RPMI and FBS 10% (Gibco Co. USA) were intraperitoneally inoculated into anesthetized mice in completely sterile conditions. Afterward, RPMI and 10% FBS were used to wash the aspirated contents of the peritoneum, and finally, the live phagocytic cells were measured with the Neubauer slide. For the OPA assay, the 100μl of serial dilutions of inactivated sera (1:2 to 1:16) were incubated with 100 μl of pneumococci for 30 min at 37°C. Then, 100 μl of phagocytic cells (1×10^6^ cells/ml) in opsonization buffer (glucose 5.56 mM, NaCl 137.93 mM, KCl 5.33 mM, CaCl_2_ 1.26 mM, MgSO_4_ 0.407 mM, MgCl_2_ 0.493 mM, KH_2_PO_4_ 0.441 mM, NaHCO_3_ 4.17 mM, Na_2_HPO_4_ 0.338 mM, and bovine gelatin 0.1%) and a complement source (fresh infant rabbit serum (4%)) were added to the immune complex microtube and incubated. Subsequently, 25μl of each reaction well was serially diluted and inoculated into the blood agar plate at two intervals (0 and 90 min). After 18-24 hours of incubation at 37°C in 5% CO2, the colony-forming unit of bacteria was measured. The assessment of antibody opsonic activity against pneumococci was calculated and compared with the control group by the following formula (23, 34, 40, 46):


$$\text{Percentage of the killed bacteria}=[1-(\frac{\text{CFU of immune serum}}{\text{CFU of pre}-\text{immune serum}})\times 100$$

### 2.10 Challenge study

To evaluate the protection against sepsis infection of pneumococcus *in vivo*, the immunized mice were infected intraperitoneally 2 weeks after the last immunization with 10^6^ CFU/mouse of pneumococcal strain ATCC49619 (serotype 19F), which is characterized by high resistance rates to several antibiotics and is one of the most common serotypes of pneumococci in Iran (47, 48). The animals were monitored for survival every 24 hours for up to 1 week. One day after the challenge, the blood of the mice was also analyzed to count the CFU/ml of pneumococci. One week post-challenge, the spleen of the mice were harvested and homogenized in 3-5 ml PBS and then serially diluted from 10^-1^ to 10^-5^. Finally, each dilution was cultured on the blood agar medium to evaluate the clearance of pneumococci from the spleen.

### 2.11 Statistical analysis

Statistical analysis was conducted using the GraphPad Prism 9 program. One and two-way analysis of variance (ANOVA) followed by Tukey’s multiple comparison tests were performed to analyze the immune responses. The Kaplan–Meier curve was used to plot the survival rate curve. All experiments were performed in triplicate and given as mean ± S.D. P-values less than 0.05 were considered statistically significant.

## 3 Results

### 3.1 Expression and purification of recombinant pneumococcal antigens

**Figure 1:**
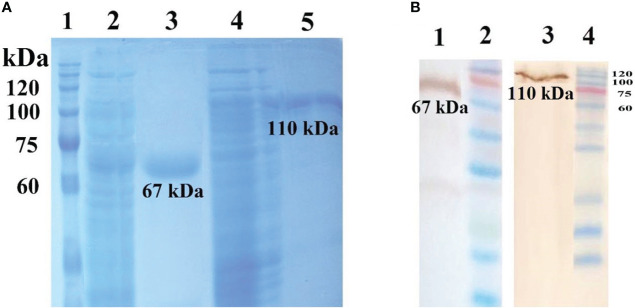
**(A)** Purification of recombinant PhtD and rPspA antigens by Ni-NTA chromatography. Lanes 1: protein ladder marker, 2: crude of rPspA, 3: purified rPspA (∼67 kDa), 4: crude of rPhtD, 5: purified rPhtD (∼110 kDa). **(B)** western blot analysis of the purified recombinant antigens using an anti-His antibody. Lanes 1: Purified rPspA (~67 kDa), 2:Protein ladder marker, 3:Purified rPhtD (~110kDa), 4: Protein ladder marker (23).

The results of the expression of recombinant rPspA and PhtD antigens with 1 mM IPTG are shown in **Figure 1**. According to the SDS-PAGE data, the size of rPspA and PhtD antigens was determined to be approximately 67 and 110 kDa, respectively. Using the 6-His tag at the C-terminus of both antigens, the recombinant antigens were purified by Ni-NTA chromatography. A single band for each purified antigen in the elution fraction is shown in **Figure 1A**. Western blotting showed that two purified recombinant antigens could react with the HRP-conjugated Anti-His tag antibody (Sigma, USA) on a nitrocellulose membrane (**Figure 1B**). These findings could confirm the expression of both recombinant PhtD and rPspA antigens. In addition, the LAL test showed an imperceptible level of LPS (<0.25 EU/ml) in the PhtD and rPspA solutions. The concentration of the purified PhtD and rPspA antigens was also 0.65 and 0.8 mg.ml^-1^, respectively.

### 3.2 Sera specific IgG elicited against rPspA and PhtD

**Figure 2:**
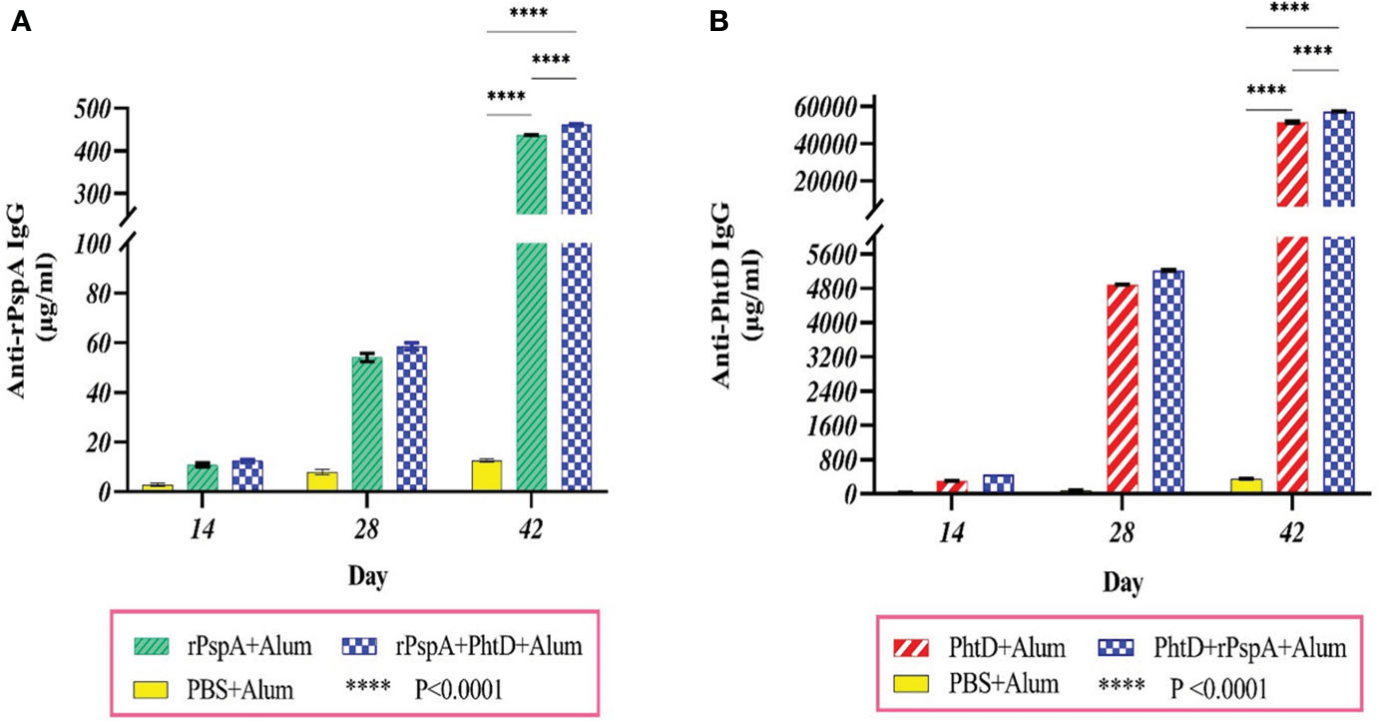
Specific sera IgG level and the boosting effect of antigen administration at different times of immunization. Groups of 7 BALB/c mice were intraperitoneally immunized on days 0, 14, and 28 with 10 μg of alum-adjuvanted rPspA alone, PhtD alone, and in combination together. The control group received PBS plus alum. Each group’s sera were collected before each administration and two weeks after the last immunization. The production of specific sera IgG against rPspA and PhtD antigens was analyzed by ELISA. **(A)** Anti-rPspA specific IgG levels after antigen administration. **(B)** Anti-PhtD specific IgG levels after antigen administration. The specific IgG responses against each antigen in the protein combination group showed a relative increase compared to the injection of each protein alone. The error bar is representative of the mean ± SD.

ELISA analysis of pooled sera from groups of mice immunized with purified antigens, either individually or in combination, showed that the production of antigen-specific IgG responses was induced remarkably. There was a significant increase between the mean serum concentration of specific IgG against PhtD or rPspA on days 14, 28, and 42 (P<0.0001), indicating the effect of a booster dose of antigen injection on increasing serum IgG antibody levels during the days post-immunization. In addition, the mean concentration of anti-rPspA sera IgG on day 42 in the immunized group with rPspA+PhtD had a relative increase compared to the group receiving rPspA alone (461.8 ± 1.7 vs. 437.4 ± 0.6 μg.ml^-1^), which was statistically significant (P<0.0001) (**Figure 2A**). Furthermore, immunization with PhtD in combination with rPspA resulted in enhanced production of anti-PhtD IgG in pooled sera compared to that of PhtD alone (57333 ± 132.6 vs. 51568.3 ± 409.6 μg.ml^-1^), which was statistically significant (P<0.0001) (**Figure 2B**). The mean concentration of each antigen-specific IgG did not decrease when the rPspA+PhtD immunization schedule was used, revealing no detectable antagonistic effect of combining both antigens. In addition, it was observed that the levels of anti-PhtD IgG antibodies were surprisingly 100 times greater than the anti-rPspA IgG, which may be due to the high immunogenicity of the PhtD antigen compared to the rPspA antigen.

### 3.3 Antigen-specific IgG1/IgG2a ratio

**Figure 3:**
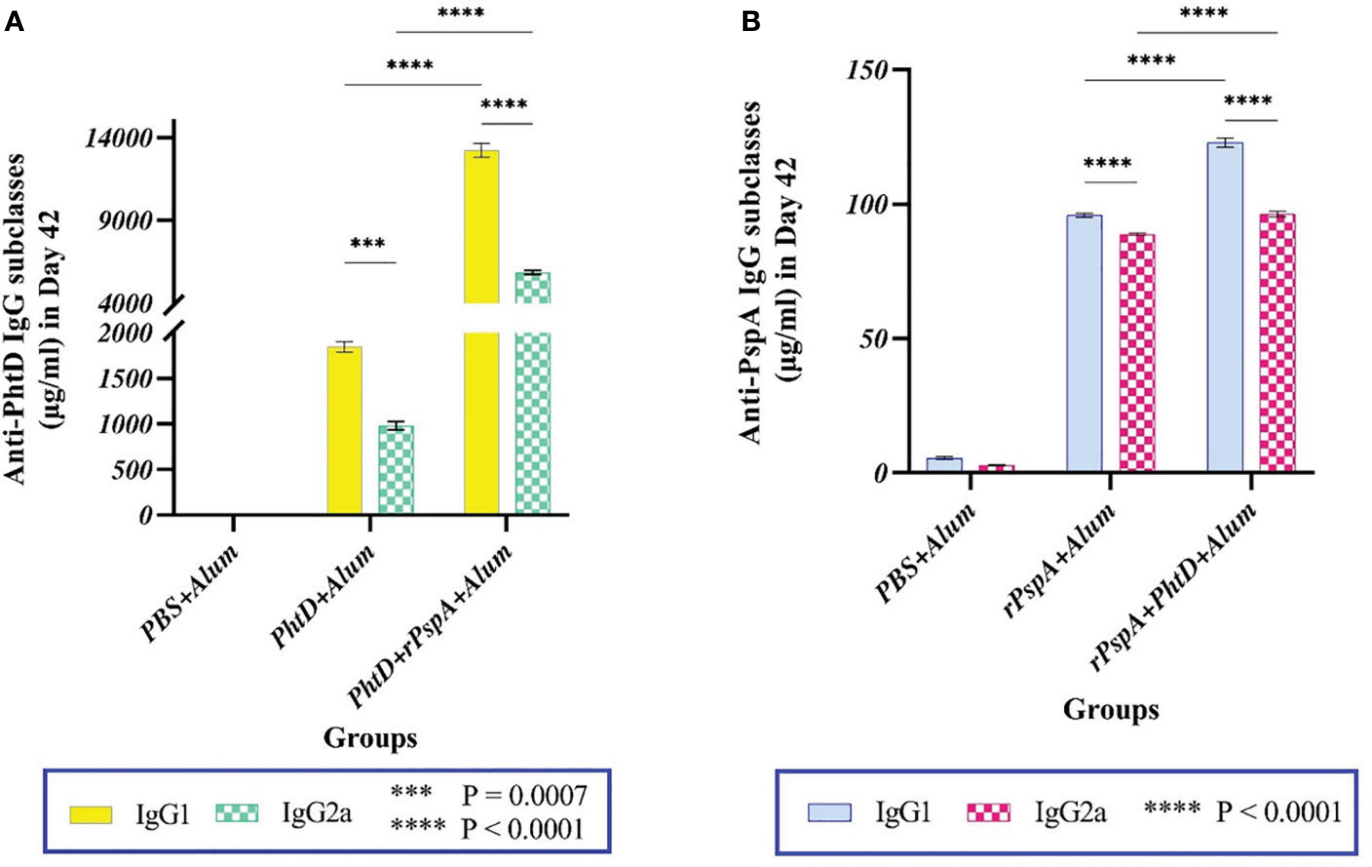
Antigen-specific serum IgG1 and IgG2a levels following intraperitoneal immunization of mice with alum-adjuvanted rPspA and PhtD alone or in combination together compared to the control group on day 42. **(A)** Co-administration of PhtD with rPspA resulted in a significant 7.16-fold increase in anti-PhtD IgG1 and an approximately 6-fold increase in specific anti-PhtD IgG2a (P<0.0001). **(B)** Co-vaccination of rPspA with PhtD resulted in a 1.3-fold increase in specific anti-rPspA IgG1 and a 1.1-fold increase in specific anti-rPspA IgG2a titers. The error bar is representative of the mean ± SD.

To evaluate the specific immune response towards humoral immunity or cellular immunity generated against each antigen, the subclasses of specific sera IgG (i.e., IgG1 and IgG2a) were investigated. The findings indicated that the administration of PhtD or rPspA proteins either alone or in combination formulations resulted in the generation of high titers of both IgG1 and IgG2a antibodies (**Figure 3**). In addition, both anti-PhtD IgG1 and IgG2a responses in the group that received rPspA+PhtD showed an increase of more than five times compared to the group that received PhtD alone, which was statistically significant (P<0.0001). In this regard, the increase in the anti-PhtD IgG1 titer in combination formulation was 7.6 times compared to the PhtD alone (13224.5 ± 413 vs. 1846.2 ± 56.73 μg.ml^-1^), and the increase in the anti-PhtD IgG2a titer in the combination formulation was 5.95 times compared to the PhtD alone (5853 ± 116 vs. 982.8 ± 46.35 μg.ml^-1^) (**Figure 3A**). The effect of combining rPspA with PhtD on increasing the level of the anti-PhtD IgG1 was more significant than increasing the level of the anti- PhtD IgG2a. Furthermore, as shown in **Figure 3B**, the increase in the anti-rPspA IgG1 titer in the combination formulation was 1.3 times compared to the rPspA alone (123 ± 1.7 vs. 96 ± 0.8 μg.ml^-1^), and the increase in the anti-rPspA IgG2a titer in the combination formulation was 1.1 times compared to the rPspA alone (96.3 ± 1vs. 89 ± 0.4μg.ml-1) (**Figure 3B**). Similar to the anti-PhtD IgG1 findings, the effect of combining PhtD with rPspA on increasing the level of anti-rPspA IgG1 was more significant than increasing the level of anti-rPspA IgG2a. Overall, the effect of rPspA on anti-PhtD IgG subclasses was greater than the effect of PhtD on the anti-rPspA IgG subclasses. The results of anti-rPspA and anti-PhtD (IgG1/IgG2a) ratios are shown in **Table 1** and indicated the elicited polarized Th-2-biased immune response to both antigens.

**Table 1:** Specific (IgG1/IgG2a) ratio against each antigen in the sera of immunized mice on day 42.

Specific IgG subclasses	Groups	(IgG1/IgG2a) ratio	Immune Response (more towards)
Anti-rPspA IgG	rPspA+Alum	1.08	Both (Humoral and Cellular) (Th-2 and Th-1)
	rPspA+PhtD+Alum	1.27	Humoral (Th-2)
Anti- PhtD IgG	PhtD+Alum	1.87	Humoral (Th-2)
	PhtD+rPspA+Alum	2.25	Humoral (Th-2)

### 3.4 Cytokine analysis

**Figure 4:**
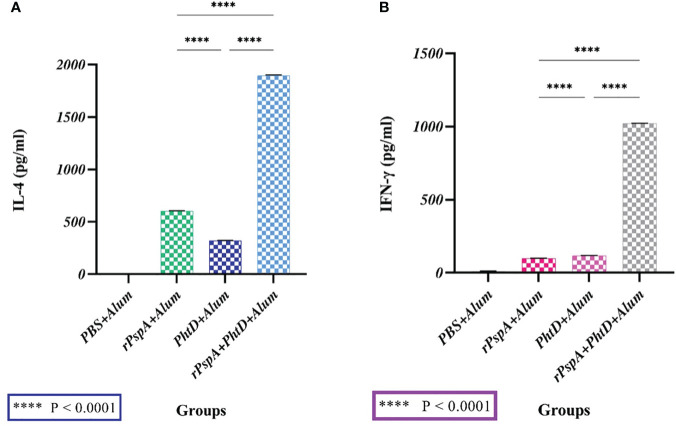
The secreted cytokine profiles from stimulated spleen cells of immunized mice with rPspA alone, PhtD alone, and rPspA plus PhtD 14 days after the last immunization *in vitro*. The cytokine profile of Th-1 (IFN-γ) and Th-2 (IL-4) was analyzed. **(A)** The mean concentration of the cytokine IL-4 in the combination group (rPspA+PhtD) showed a significant increase compared to the rPspA-alone and PhtD-alone groups, respectively (P<0.0001). **(B)** The mean concentration of the cytokine IFN-γ in the combination group (rPspA+PhtD) also showed a significant increase compared to the rPspA-alone and PhtD-alone groups, respectively (P<0.0001). The error bar is representative of the mean ± SD.

We evaluated IL-4 and IFN-γ secretion levels from the spleen lymphocyte cells of immunized mice stimulated with rPspA alone, PhtD alone, and rPspA+PhtD *in vitro*. As shown in **Figure 4**, each antigen alone or in combination formulations could induce a mixture of Th-1 and Th-2 immune responses, although more towards Th-2, and the production of both cytokines was induced remarkably. In **Figure 4A**, the mean concentration of IL-4 in the immunized groups with rPspA, PhtD, and rPspA+PhtD were 602.7 ± 2.5, 322.3 ± 2.5, and 1896.3 ± 5.5 pg.ml^-1^, respectively. The level of IL-4 in the immunized group with rPspA+PhtD showed a significant increase compared to the level of IL-4 in the immunized groups with each antigen rPspA or PhtD alone (3.14- and 5.9-fold, respectively) (P<0.0001). In addition, in **Figure 4B**, the mean concentration of IFN-γ in the immunized groups with rPspA, PhtD, and rPspA+PhtD was 98.3 ± 2, 117.3 ± 2.5, and 1022 ± 1.7 pg.ml^-1^, respectively. The level of IFN-γ in the rPspA+PhtD group showed a significant increase compared to the level of IFN-γ in each antigen rPspA or PhtD alone groups (10.43- and 8.8-fold, respectively) (P<0.0001). Finally, the IL-4/IFN-γ ratio in the combination group was 1.85. The high level of IL-4 compared to IFN-γ showed confirmation of the immune response pathway direction more toward humoral immunity (Th-2).

### 3.5 Serum bactericidal activity

**Figure 5:**
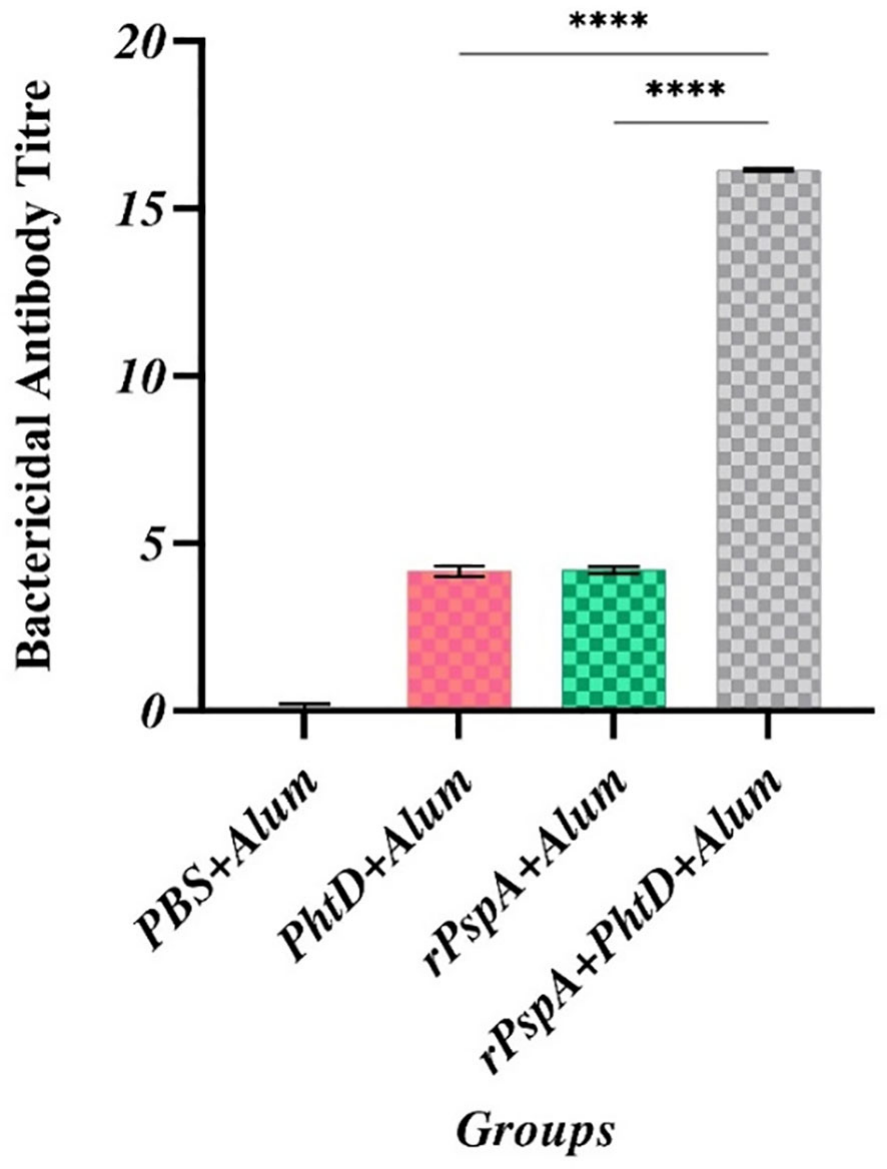
Comparative analysis of complement-mediated killing feature of anti-PhtD and anti-rPspA antibodies against pneumococcus strain ATCC49619. In the titer mentioned, serum antibodies can kill 50% of the pneumococci compared to the control group following complement cell activation and pore formation on the surface of pneumococci. ****: p<0.0001.

The SBA assay was performed to assess the complement-mediated killing features of anti-rPspA and anti-PhtD antibodies against pneumococcal strain ATCC 49619 (serotype 19F). The titer of antibody in serum on day 42 that required the killing of 50% of the bacteria by complement activation was obtained at the dilutions of 1:4, 1:4, and 1:16 in the rPspA-alone, PhtD-alone, and combination groups, respectively, showing that all three immunized groups could activate humoral immunity. In addition, as shown in **Figure 5**, the highest serum bactericidal activity was observed in the group immunized with rPspA+PhtD, indicating that co-immunization of rPspA with PhtD led to an increase (2-fold) in the bactericidal activity of antibodies against pneumococci. The rPspA-alone and PhtD-alone immunized groups had no significant differences. No complement-mediated killing activity was observed in the presence of PBS as a control group.

### 3.6 Opsonophagocytic killing activity

**Figure 6:**
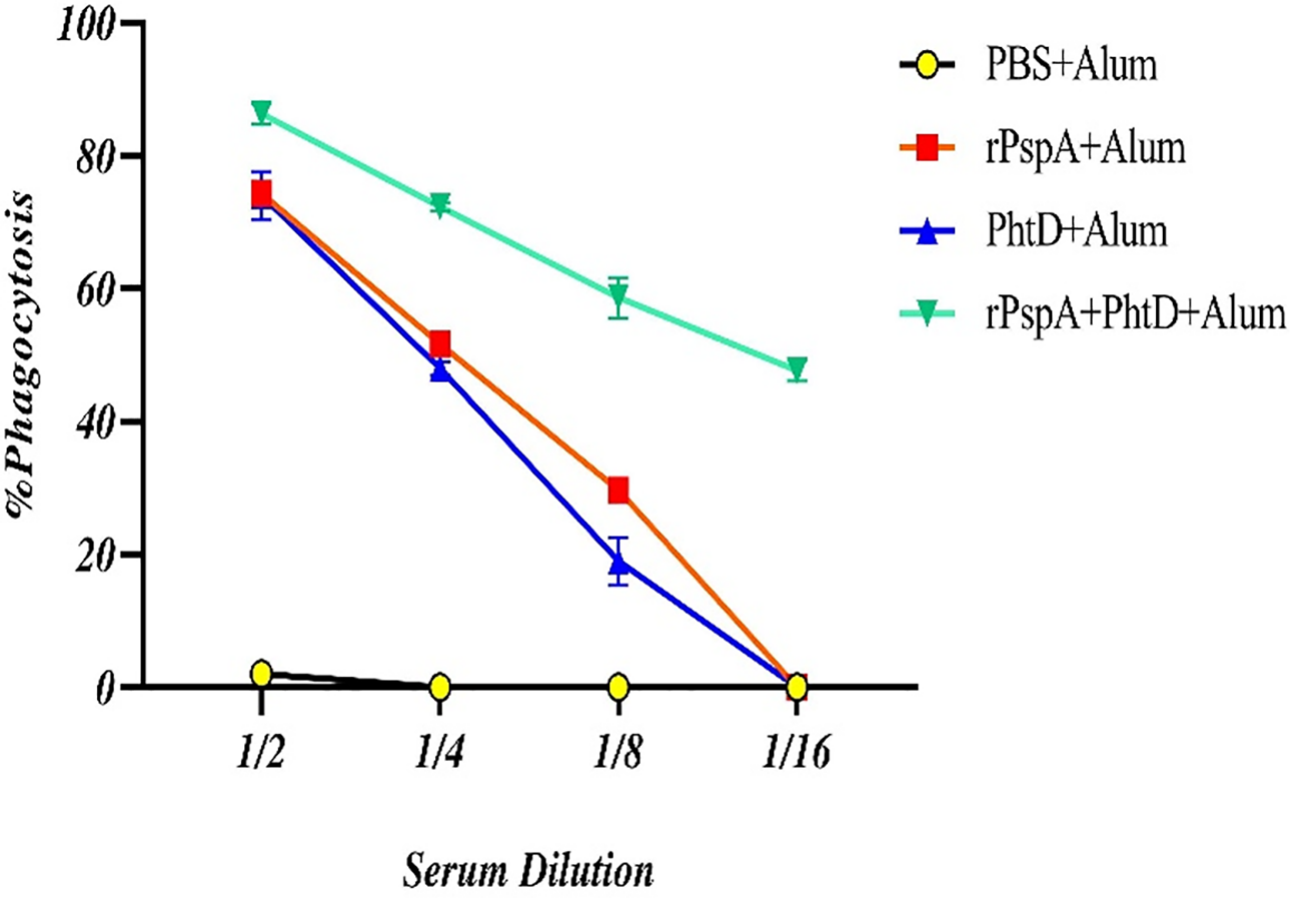
Comparative analysis of the opsonophagocytosis-mediated killing activity of anti-PspA and anti-PhtD IgG antibodies. The opsonized pneumococci with serial dilutions of sera from each group were exposed to the peritoneal phagocytic cells and the complement source, resulting in the facilitation of phagocytosis and a reduction in the number of pneumococci compared to the control group. The mice that were immunized with rPspA in combination with the PhtD showed more effective opsonic activity of the antibodies than those immunized with antigens alone.

The OPA assay was accomplished as a gold-standard test for evaluating the efficiency and effectiveness of pneumococcal vaccines. In our OPA assay, the sera of four immunized groups were assessed on day 42 at dilutions ranging from 1:2 to 1:64 to calculate the percentage of phagocytosis of the opsonized pneumococci. The phagocytic cells can scavenge pneumococci opsonized with anti-rPspA and anti-PhtD antibodies more rapidly in the presence of the complement system following the expression of its Fc-receptors, which attach to the Fc fragment of antibodies and CR1-receptors that bind to the C3b fragment of the complement system. As shown in **Figure 6**, the phagocytic-mediated killing activity at a serum dilution of 1:2 was calculated to be 86.3%, 74.3%, and 74% in the sera of the rPspA+PhtD, rPspA alone, and PhtD alone immunized groups, respectively. The sera of the co-immunization of the rPspA plus PhtD group still showed an opsonophagocytic activity of up to 48-49% at a dilution of 1:16. These data indicated that the antibodies raised against rPspA+PhtD co-administration in all dilutions act as a good opsonic for the killing of pneumococci and were more effective than other groups.

### 3.7 Bacterial clearance and survival rate

**Figure 7:**
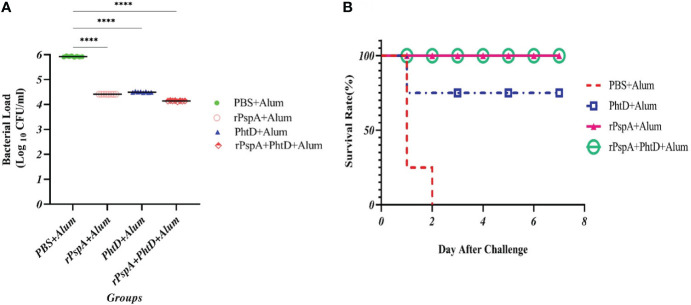
*In vivo* functional activity of raised specific antibodies in immunized mice after intraperitoneal pneumococcal challenge. **(A)** Pneumococcal microbial load in blood samples from each group in terms of (CFU/ml) 24 hours after the bacterial challenge. The specific antibodies significantly decreased the bacterial load in the blood compared to the control group. **(B)** Kaplan–Meier survival curve in immunized mice groups after intraperitoneal challenge with *S. pneumoniae* ATCC 49619 (serotype 19F). ****: p<0.0001.

To assess the protection elicited by immunization with recombinant rPspA and PhtD, either alone or in combination, BALB/c mice were challenged with *S. pneumoniae* ATCC 49619 (serotype 19F) at a dose of two logs_10_ higher than the 50% lethal dose. The bacterial burden was then measured at 24 hours post-challenge in the blood of immunized mice and showed that the immunized mice with rPspA, rPhtD, or rPspA+PhtD could clear and reduce the pneumococci to less than 40000 CFU/ml in the blood. This decrease in the bacterial load was 25 to 100-fold compared to the control group (p< 0.0001) (**Figure 7A**). In addition, the survival rate results showed that all control mice died after 48 hours. In the immunized group with PhtD, one mouse died after 24 hours. The survival rate for rPspA protein was 100% (**Figure 7B**). The mice receiving the combination of rPspA and PhtD survived significantly longer than those receiving PhtD alone or PBS. Finally, the results of spleen clearance from the pneumococcal infection 1 week after the challenge indicated that pneumococcal clearance was 100% in the mice immunized with the protein combination and no CFU/ml were counted on blood agar plates (data not shown).

## 4 Discussion

Extensive efforts have been made to introduce various effective platforms for pneumococcal vaccination, focusing on evaluating the potential of multivalent conserved protein-based vaccines. Even though many pneumococcal protein vaccines have been attempted with good animal data, they have failed in human studies. However, the main benefit of using conserved proteins is overcoming the limitations of pneumococcal polysaccharide-based vaccines, e.g., the coverage of all pneumococcal serotypes (as serotype-independent candidates) and the induction of the potent T-lymphocyte-dependent immune responses in children younger than 2 years old and adults older than 65 years old (8, 9, 49, 50).

In our previous study, we designed a novel epitope-based, serotype-independent rPspA vaccine candidate consisting of the immunodominant crass-reactive truncated domain of PspA clades 1 to 5 and three PspA proline-rich motif groups to cover clade-dependent immunity against PspA Families 1 and 2 (23). Moreover, our immunoinformatics findings exhibited that this designed protein can be one of the best putative PspA-based pneumococcal vaccine candidates and that it is valuable for immunological evaluation (23). In the present study, we sought to evaluate the protective response of the combination of this new rPspA construct with PhtD as an attractive pneumococcal vaccine candidate against invasive pneumococcal infection and analyze the effect of PhtD on specific anti-rPspA immune responses when applied in a combination formulation and vice versa. Researchers have focused on two models of non-invasive and invasive pneumococcal infections. Although most pneumococcal infections affect the respiratory tract through pneumonia as a non-invasive infection, the mortality and morbidity rates in patients with developed bacteremia and sepsis, which model as invasive infections, are very high (3, 51, 52). Therefore, we decided to assess the effectiveness of our proposed bivalent vaccine candidate against the pneumococcal sepsis infection model. The use of these two antigens as candidates for a bivalent pneumococcal vaccine has not been investigated so far. In addition, we used the intraperitoneal route for the pneumococcal challenge to achieve the sepsis model of pneumococcal infection (as one of the invasive infections). Although the usual route of the pneumococcal entrance is the nasal route, according to the reported protocol for “Animal Models of *Streptococcus pneumoniae* Disease” conducted by 53, “Mice sepsis model can be generated by either the Intraperitoneal (i.p.) or the Intravenous (i.v.) route of infection. Sepsis can also develop secondary to pneumonia induced by Intranasal/Intratracheal (i.n./i.t.) routes or to meningitis following intracranial injection. However, the kinetics of bacterial transit from the lungs or brain into the bloodstream cannot be controlled experimentally. Therefore, performing viable counts in the blood after i.n./i.t. or intracranial inoculations does not constitute a direct measure of bacterial clearance operating in those districts, and it is not the finest approach to evaluate immune mechanisms and efficacy of drugs or vaccines in experimental pneumonia or meningitis. As we have mentioned above, the i.v. route is a direct and valuable system for inducing infection in the bloodstream and studying the mechanisms of bacterial clearance. However, the technique can be time-consuming and difficult to perform compared to i.p. infection” (53). “Besides that i.p. inoculation is technically easier, Briles et al. observed that certain pneumococcal strains that were lethal following injection of mice by the i.p. route were avirulent by the i.v. route, indicating that i.p.-induced sepsis is much more severe (54, 55). For this reason, the i.p. route is widely used to mouse passage pneumococci in order to render them more virulent” (53). Therefore, we used the i.p. route for a making pneumococcal sepsis infection model in immunized mice.

In this study, our findings demonstrated that co-immunization of the new rPspA construct with PhtD evoked high levels of anti-rPspA and anti-PhtD specific IgGs. There was a synergistic effect of the combination of both antigens on antigen-specific IgG compared to each antigen alone. These data are in accordance with previous studies that showed that there was no apparent decrease in antigen-specific antibody titers when the pneumococcal antigens were applied in combination with each other (37, 38, 42, 56, 57). In this context, Ogunni et al. demonstrated that using multiple proteins (for example, combination formulations of PdB+PspA, PhtB+PhtE, PdB+PhtB, PdB+PspC, PspA+PhtB, and PdB+PspA+PhtB) were often found to have a synergistic or additive effect, even when the individual proteins had little value on their own (38, 56). As both proteins contribute to different stages of the pathogenic process, they may have complementary or synergistic effects (38). However, combining several antigens with different roles in bacterial pathogenicity does not always lead to an increase or change in the specific-antigen antibody titer (38).

In the next step, we examined the production of the antigen-specific IgG subclasses, IgG1 and IgG2a, to determine the Th-2 and Th-1 polarized responses, respectively. These two antigens were able to significantly induce both antigen-specific IgG1 and IgG2a isotypes in their formulations. However, IgG1 was predominant, suggesting that the two antigens developed a strong polarized Th-2-biased immune response. One of the possible reasons could be the use of alum adjuvant in this study. Pneumococcus is an extracellular pathogen, and a powerful Th-2 response can be effective against its infection. In addition, it has been reported that alum adjuvant stimulates the Th-2 immune response. As alum has been commonly used as an adjuvant in clinical trials and pneumococcal vaccine studies conducted over the past decade (8, 58, 59), we used alum adjuvant in this study. However, this polarized Th-2 response may depend on the nature of the antigens and the immunological pathway, which can stimulate such a response. Malekan et al. reported that the PhtD antigen could induce a dominant Th-2 immune response (36, 60), and Kothari et al. (61) and Tada et al. (41) showed that the PspA families predominantly produced IgG1. Our results were similar to these reports. However, the immunization of mice with single or combination formulations of rPspA or PhtD without alum adjuvant presence or in the presence of other adjuvants is required to determine the role of each antigen or adjuvant in immune responses. Furthermore, other antigen-specific IgG subclasses, such as IgG2b, IgG2c, or IgG3, are suggested to be investigated in future studies.

Considering that the Th-2 immune response plays a vital role in eliminating pneumococcal infection, this increase in the antigen-specific IgG1 titer may be more effective in generating a protective response against pneumococcal systemic infections than individual groups. Among the three formulations (rPspA, PhtD, and rPspA+PhtD), the immunization plan with the rPspA+PhtD formulation was successful and satisfactory. However, we also observed a high titer of Th-1 responses, which may be more effective in activating macrophage and dendritic cells and strengthening innate immunity against pneumococcal infections. A probable reason behind this might be the fact that PhtD or rPspA may bind to toll-like receptors (TLRs). Although no studies have been reported on the binding of the PhtD or PspA proteins to TLRs so far, it has been proven that some pneumococcal proteins, such as pneumolysin or heat shock protein 40 (DnaJ), are considered toll-like receptor (TLR) agonists, which have mucosal and subcutaneous adjuvant functions (62). These proteins are capable of inducing T1 immune responses through binding to toll-like receptors 4 or 2 and the activation of the MAPKs, NF-κB, and PI3K-Akt pathways, which lead to the activation of bone marrow-derived dendritic cells and macrophages (62–64). In addition, in this study, we investigated only the levels of IFN-γ cytokines, representing the Th-1 immune pathway. As a result, whether PhtD or rPspA proteins can bind to toll-like receptors to activate the immune pathways or other cytokine profiles can play a role in stimulating the Th-1 immune pathways and how this raised Th-1 response can be effective in creating protective immunity against pneumococcal infections requires more studies in the future.

Moreover, surprisingly, we found that the effect of rPspA on anti-PhtD IgG subclasses was greater than the effect of PhtD on the anti-rPspA IgG subclasses. Both the anti-PhtD IgG1 and IgG2a responses in the group that received rPspA+PhtD showed an increase of more than five times compared to the group that received PhtD alone. The anti-PhtD (IgG1/IgG2a) ratio was increased from 1.87 to 2.25, while the anti-PspA (IgG1/IgG2a) ratio was increased from 1.08 to 1.27 (in the groups that received the proteins alone vs. combination formulations, respectively). In this study, we only investigated the phenomenon of antigen-specific IgG subclasses in the immunized groups with combination formulation compared to each antigen alone. We do not know the exact cause of this phenomenon. However, it may be due to the newly designed rPspA structure. This construct is not a natural structure of full-length PspA, however, it has immunodominant B- and T-cell epitopes with high immunogenicity scores from cross-reactive truncated regions of five PspA clades (23). As we had reported previously (23), immune simulation servers predicted that this construct would contain antigenic regions with a powerful potential to induce both humoral and cellular immune pathways. The presence of a repeated number of high immunogenic PspA epitopes stimulating both arms of the immune system can have a more significant synergistic effect than the full-length structure of PhtD, which may suggest that not all parts of PhtD stimulate the immune system. As a result, PhtD cannot lead to a 5-fold increase in anti-rPspA responses as rPspA can. However, what has been reported so far about these two proteins in previous studies is the mechanism of the two proteins in the escape of pneumococcus from the immune system. PhtD can reduce the complement deposition on the pneumococcal surface, and PspA, in addition to hindering the deposition of the complement on the surface, leads to the blocking of lactoferrin’s bactericidal activity. But no studies have reported on protein signaling pathways stimulating the immune system alone or in combination with any protein so far. As a result, more studies are warranted to clarify why the effect of rPspA is greater than the effect of PhtD on the antigen-specific IgG subclasses.

To confirm the Th-2 and Th-1 polarized responses, we then investigated the levels of IL-4 and IFN-γ cytokines secreted from the spleen lymphocytes of the immunized groups. Our findings showed that the levels of IL-4 and IFN-γ cytokines were consistent with the results of IgG1 and IgG2a. In other words, the increase in the IgG subclasses titer in the combination group compared to the single group may be due to an increase in the induction of the IL-4 and IFN-γ cytokine secretion. It has been proven that IL-4 has many biological roles, such as naïve T cell differentiation to Th-2 and B-cells differentiation to plasma cells (65), as well as a Th-1 response characterized by increased production of IFN-γ and IgG2a (66, 67). In an immunoinformatics study, we reported that our designed PspA_1-5c+p_ was predicted to have the IFN-γ inducing MHC class II binding peptides and potency of the IL-4 cytokine induction (23). In the current study, we proved that this construct induces both humoral and cellular immune pathways. Overall, these results also show the success of the bioinformatics design of our rPspA vaccine candidate.

Next, to clarify whether the elevated specific anti-PspA and anti-PhtD antibodies can mediate immune protection against pneumococcal infection or not, we performed two *in vitro* functional antibody tests. Complement-mediated killing activity of anti-PspA and anti-PhtD demonstrated that the highest bactericidal activity was detected at a 1:16 dilution of combined group sera to kill more than 50% of pneumococci compared to the control group, while sera of single antigen groups had bactericidal activity at a dilution of 1:4. Moreover, the combination of the rPspA with PhtD resulted in a promotion of the opsonophagocytic activity from 74% in single group sera to 86.3% at a 1:2 dilution. The increase in bactericidal activity from 1:4 in single groups to 1:16 in the combined group and the increase in the OPA show that not only were the antibodies titers high in the antigen combination group, but also these antibodies showed more functional activities compared to antigen alone group sera. A possible explanation for this is that both PhtD and rPspA have a common role in inhibiting C3 deposition of the complement system on the surface of pneumococci (8, 9, 31, 49, 68), so co-immunization of two antigens could strengthen OPA activity and lead to a synergistic effect.

Even though the OPA and SBA tests proved the functionality of IgG antibodies, a question that was raised is whether the function of the antibody can be also verified under physiological conditions. For this purpose, the challenge of the immunized groups was done with pneumococci as a systemic infection model, and the microbial load in the blood, bacterial clearance from the spleen, and the survival rate of the mice after the challenge were investigated. The results showed that the combination of PhtD with rPspA provided higher degrees of protection than each protein alone. The *in vivo* result corroborated the *in vitro* OPA and SBA results. PspA and PhtD have been reported to inhibit the recognition of pneumococci by the host’s complement system (31, 41). Furthermore, pneumococci can also escape from killing by lactoferrin with the help of their PspA as a specific receptor for lactoferrin (69). According to the literature, “Lactoferrin (LF) or lactotransferrin is an iron-sequestering glycoprotein that is produced by the body itself, as a secretion by exocrine glands (such as maternal milk or tears) and secondary granules of human neutrophils, it can also be taken as a supplement, where it then acts a nutraceutical or functional food. It has important immunological properties and is both antibacterial and antiviral. It also binds iron and is transferred via a variety of receptors into and between cells, serum, bile, and cerebrospinal fluid.” (70). Besides, in our previous *in silico* study, we demonstrated that “the designed PspA_1-5c+p_ construct could be attached to human lactoferrin molecule effectively via both regions representing PspA Family 1 and 2 in construct with the lowest energy binding of and -1128.9 and -987.2 kcal.mol^-1^ “(23). “Senkovich et al. also suggested that inhibition of this interaction using small molecules or antibodies may permit the lactoferrin natural bactericidal effect to preserve the host from pneumococcal colonization and infection and can use for designing therapeutic strategies for the prevention or treatment of pneumococcal diseases” (71). Since the route of the bacterial challenge is the intraperitoneal route, it is not far-fetched to expect pneumococcus to reach different parts of the body through systemic infection, wherever lactoferrin may be present. Therefore, a reason behind the elevated protective immune responses to invasive pneumococcal infections in the group of mice immunized with a combination of rPspA with PhtD might be the production of a high level of IgG antibodies compared to antigen alone and the synergistic effect of anti-PhtD and anti-PspA specific IgGs to prevent both antigen-mediated inhibitory functions of the host’s complement system and lactoferrin.

In addition, our findings were also in accordance with previous studies that reported that the combinations PspA+PdB (56), PdB+PspC (38), PspA+PspC (38), PspA+ClpP (37), ClpP+Ply+LpI (57), PhtD+PcpA+PlyD1 (72), and a combination of ClpP, DnaJ, and GroEL (42) showed the highest protection with longer survival rates than single antigen administration. In general, the multivalent protein-based vaccine candidate formulations have shown a higher protective response than the monovalent vaccine formulations so far. This increase in the protective immune response of multivalent antigen formulations is probably due to the complementary or synergistic role of antigens in pneumococcal pathogenesis, leading to a subsequent increase in the production and secretion of the cytokines and opsonic antibodies following activation of both Th-2 and Th-1 immune responses in antigen combination groups. In addition, it is reported that the antibody against the N-terminal of the PhtD protein exhibits cross-reactivity with the proline-rich region of the PspA protein due to the similarity of their amino acid sequence (46). Our PspA construct (PspA_1-5c+p_) is a chimeric protein and contains three PspA proline-rich motif groups (23). Although our designed PspA protein, in terms of the sequence of the proline-rich region and also structurally, is different from the native pneumococcal PspA structure, there is the possibility of cross-reactivity with the PhtD protein. Therefore, the elevated protective immune response in the combined group, compared to the individual groups, may be attributed to cross-reaction or cross-protection. Consequently, a potential future research direction could involve investigating the cross-reaction and cross-protection of anti-PspA antibodies against PhtD and vice versa.

## 5 Conclusion

In this article, we offer a bivalent pneumococcal protein-based vaccine candidate comprising novel rPspA and PhtD that significantly enhances protective humoral immunity against pneumococcal systemic infection in mice. In this research, we also sought to investigate the effect of PhtD on the response of rPspA and vice versa. We observed that the combination of PhtD with rPspA produced a synergistic effect on the specific IgG and increased the anti-rPspA IgG1 response by 1.3 times. rPspA also increased the level of anti-PhtD IgG1 by 7.6 times. Although both antigens were capable of eliciting a mixture of Th-1 and Th-2 immune responses, the Th-2 immune response was dominant. Finally, since the Th-2 immune response plays an important role in eliminating pneumococcal infection, this increase in antigen-specific IgG and IgG1 titers could be more effective in OPA, SBA, bacterial clearance, and survival rate of mice immunized with both antigens than each antigen alone. However, further studies are needed to investigate the safety and durability of the proposed bivalent vaccine candidate, as well as other immunization routes and models of infection, using other adjuvants, checking the levels of other subclasses of antibodies or cytokines, the level of each cytokine separately against each antigen in the combination formulation, cellular signaling pathways, flow cytometric studies, and the combination of our novel rPspA with other pneumococcal proteins. In addition, the role of the cellular arm in the immune response (Th-1 immune response) against pneumococcal infection should be investigated. Furthermore, since the retaining and presence of His tag can influence the immunogenicity of recombinant proteins, therefore, careful consideration is needed before use in any step of animal study and clinical trials. 

## Data availability statement

The original contributions presented in the study are included in the article/supplementary material. Further inquiries can be directed to the corresponding author.

## Ethics statement

All animals were kept and handled in accordance with the ethical guidelines of the Institutional Animal Care and Use Committee at the Pasteur Institute of Iran (Approval ID: IR.PII.REC.1398.005). The study was conducted in accordance with the local legislation and institutional requirements.

## Author contributions

Conceptualization: SM, RA, and EA; methodology: EA, and MS; validation: SM, RA; formal analysis: SM, RA, MS, and EA; investigation: EA, and MS; writing-original draft preparation: EA; writing-review and editing: SM, RA, and EA; visualization: EA, MS; project administration: SM and RA; Resources: SM and EA; Supervision: SM. All authors contributed to the article and approved the submitted version.

## References

[1] Chen A Mann B Gao G Heath R King J Maissoneuve J. Multivalent pneumococcal protein vaccines comprising pneumolysoid with epitopes/fragments of CbpA and/or PspA elicit strong and broad protection. Clin Vaccine Immunol (2015) 22:1079–89. doi: 10.1128/CVI.00293-15 PMC4580740 26245351

[2] Chen X Li B Yu J Zhang Y Mo Z Gu T. Comparison of four adjuvants revealed the strongest protection against lethal pneumococcal challenge following immunization with PsaA-PspA fusion protein and AS02 as adjuvant. Med Microbiol Immunol (2019) 208:215–26. doi: 10.1007/s00430-019-00579-9 30707297

[3] Demirdal T Sen P Emir B. Predictors of mortality in invasive pneumococcal disease: a meta-analysis. Expert Rev Anti-infective Ther (2021) 19:927–44. doi: 10.1080/14787210.2021.1858799 33382642

[4] Mousavi SF Nobari S Ghezelgeh FR Lyriai H Jalali P Shahcheraghi F. Serotyping of Streptococcus pneumoniae isolated from Tehran by Multiplex PCR: Are serotypes of clinical and carrier isolates identical? Iranian J Microbiol (2013) 5:220. PMC3895558 24475327

[5] van Aalst M Lötsch F Spijker R Van Der Meer JT Langendam MW Goorhuis A. Incidence of invasive pneumococcal disease in immunocompromised patients: a systematic review and meta-analysis. Travel Med Infect Dis (2018) 24:89–100. doi: 10.1016/j.tmaid.2018.05.016 29860151

[6] Feldman C Anderson R. Current and new generation pneumococcal vaccines. J Infection (2014) 69:309–25. doi: 10.1016/j.jinf.2014.06.006 24968238

[7] Brooks WA Chang L-J Sheng X Hopfer R Team PS. Safety and immunogenicity of a trivalent recombinant PcpA, PhtD, and PlyD1 pneumococcal protein vaccine in adults, toddlers, and infants: A phase I randomized controlled study. Vaccine (2015) 33:4610–7. doi: 10.1016/j.vaccine.2015.06.078 26143615

[8] Lagousi T Basdeki P Routsias J Spoulou V. Novel protein-based pneumococcal vaccines: assessing the use of distinct protein fragments instead of full-length proteins as vaccine antigens. Vaccines (2019) 7:9–27. doi: 10.3390/vaccines7010009 30669439 PMC6466302

[9] Converso T Assoni L André G Darrieux M Leite LCDC. The long search for a serotype independent pneumococcal vaccine. Expert Rev Vaccines (2020) 19:57–70. doi: 10.1080/14760584.2020.1711055 31903805

[10] Oliveira GS Oliveira MLS Miyaji EN Rodrigues TC. Pneumococcal vaccines: past findings, present work, and future strategies. Vaccines (2021) 9:1338–54. doi: 10.3390/vaccines9111338 PMC8620834 34835269

[11] Huang J Gingerich AD Royer F Paschall AV Pena-Briseno A Avci FY. Broadly reactive human monoclonal antibodies targeting the pneumococcal histidine triad protein protect against fatal pneumococcal infection. Infection Immun (2021) 89:e00747–20. doi: 10.1128/IAI.00747-20 PMC8091081 33649050

[12] André GO Borges MT Assoni L Ferraz LF Sakshi P Adamson P. Protective role of PhtD and its amino and carboxyl fragments against pneumococcal sepsis. Vaccine (2021) 39:3626–32. doi: 10.1016/j.vaccine.2021.04.068 34045100

[13] Hammitt LL Campbell JC Borys D Weatherholtz RC Reid R Goklish N. Efficacy, safety and immunogenicity of a pneumococcal protein-based vaccine co-administered with 13-valent pneumococcal conjugate vaccine against acute otitis media in young children: A phase IIb randomized study. Vaccine (2019) 37:7482–92. doi: 10.1016/j.vaccine.2019.09.076 31629570

[14] Bologa M Kamtchoua T Hopfer R Sheng X Hicks B Bixler G. Safety and immunogenicity of pneumococcal protein vaccine candidates: Monovalent choline-binding protein A (PcpA) vaccine and bivalent PcpA–pneumococcal histidine triad protein D vaccine. Vaccine (2012) 30:7461–8. doi: 10.1016/j.vaccine.2012.10.076 23123106

[15] Darrieux M Moreno AT Ferreira DM Pimenta FC De Andrade ALS Lopes AP. Recognition of pneumococcal isolates by antisera raised against PspA fragments from different clades. J Med Microbiol (2008) 57:273–8. doi: 10.1099/jmm.0.47661-0 18287288

[16] Mukerji R Mirza S Roche AM Widener RW Croney CM Rhee D-K. Pneumococcal surface protein A inhibits complement deposition on the pneumococcal surface by competing with the binding of C-reactive protein to cell-surface phosphocholine. J Immunol (2012) 189:5327–35. doi: 10.4049/jimmunol.1201967 PMC3517878 23105137

[17] Ren B Li J Genschmer K Hollingshead SK Briles DE. The absence of PspA or presence of antibody to PspA facilitates the complement-dependent phagocytosis of pneumococci in vitro. Clin Vaccine Immunol (2012) 19:1574–82. doi: 10.1128/CVI.00393-12 PMC3485889 22855389

[18] Mirza S Benjamin WH Jr Coan PA Hwang S-A Winslett A-K Yother J. The effects of differences in pspA alleles and capsular types on the resistance of *Streptococcus pneumoniae* to killing by apolactoferrin. Microbial Pathogenesis (2016) 99:209–19. doi: 10.1016/j.micpath.2016.08.029 27569531

[19] Hollingshead SK Becker R Briles DE. Diversity of PspA: mosaic genes and evidence for past recombination in *Streptococcus pneumoniae* . Infection Immun (2000) 68:5889–900. doi: 10.1128/IAI.68.10.5889-5900.2000 PMC101551 10992499

[20] Daniels CC Coan P King J Hale J Benton KA Briles DE. The proline-rich region of pneumococcal surface proteins A and C contains surface-accessible epitopes common to all pneumococci and elicits antibody-mediated protection against sepsis. Infection Immun (2010) 78:2163–72. doi: 10.1128/IAI.01199-09 PMC2863538 20194601

[21] Shafaghi M Bahadori Z Madanchi H Ranjbar MM Shabani AA Mousavi SF. Immunoinformatics-aided design of a new multi-epitope vaccine adjuvanted with domain 4 of pneumolysin against Streptococcus pneumoniae strains. BMC Bioinf (2023) 24:1–27. doi: 10.1186/s12859-023-05175-6 PMC9951839 36829109

[22] Jedrzejas MJ Hollingshead SK Lebowitz J Chantalat L Briles DE Lamani E. Production and characterization of the functional fragment of pneumococcal surface protein A. Arch Biochem Biophysics (2000) 373:116–25. doi: 10.1006/abbi.1999.1544 10620330

[23] Afshari E Cohan RA Sotoodehnejadnematalahi F Mousavi SF. In-silico design and evaluation of an epitope-based serotype-independent promising vaccine candidate for highly cross-reactive regions of pneumococcal surface protein A. J Trans Med (2023) 21:13. doi: 10.1186/s12967-022-03864-z PMC9830136 36627666

[24] Nabors GS Braun PA Herrmann DJ Heise ML Pyle DJ Gravenstein S. Immunization of healthy adults with a single recombinant pneumococcal surface protein A (PspA) variant stimulates broadly cross-reactive antibodies to heterologous PspA molecules. Vaccine (2000) 18:1743–54. doi: 10.1016/S0264-410X(99)00530-7 10699322

[25] Darrieux M Miyaji EN Ferreira D Lopes L Lopes APY Ren B. Fusion proteins containing family 1 and family 2 PspA fragments elicit protection against *Streptococcus pneumoniae* that correlates with antibody-mediated enhancement of complement deposition. Infection Immun (2007) 75:5930–8. doi: 10.1128/IAI.00940-07 PMC2168346 17923518

[26] Melin M Coan P Hollingshead S. Development of cross-reactive antibodies to the proline-rich region of pneumococcal surface protein A in children. Vaccine (2012) 30:7157–60. doi: 10.1016/j.vaccine.2012.10.004 23072893

[27] Mukerji R Hendrickson C Genschmer KR Park S-S Bouchet V Goldstein R. The diversity of the proline-rich domain of pneumococcal surface protein A (PspA): Potential relevance to a broad-spectrum vaccine. Vaccine (2018) 36:6834–43. doi: 10.1016/j.vaccine.2018.08.045 PMC6366627 30293761

[28] Briles D Paton J Mukerji R Swiatlo E Crain M. Pneumococcal vaccines. Microbiol Spectr (2019) 7:7.6. 2. doi: 10.1128/microbiolspec.GPP3-0028-2018 PMC10921951 31858954

[29] Roberts S Williams CM Salmon SL Bonin JL Metzger DW Furuya Y. Evaluation of Pneumococcal Surface Protein A as a Vaccine Antigen against Secondary *Streptococcus pneumoniae* Challenge during *Influenza A* Infection. Vaccines (2019) 7:146–55. doi: 10.3390/vaccines7040146 PMC6963301 31614565

[30] Piao Z Akeda Y Takeuchi D Ishii KJ Ubukata K Briles DE. Protective properties of a fusion pneumococcal surface protein A (PspA) vaccine against pneumococcal challenge by five different PspA clades in mice. Vaccine (2014) 32:5607–13. doi: 10.1016/j.vaccine.2014.07.108 25132335

[31] Scott NR Mann B TuOmanen EI Orihuela CJ. Multi-valent protein hybrid pneumococcal vaccines: A strategy for the next generation of vaccines. Vaccines (2021) 9:209–25. doi: 10.3390/vaccines9030209 PMC8002124 33801372

[32] Akbari E Negahdari B Faraji F Behdani M Kazemi-Lomedasht F Habibi-Anbouhi M. Protective responses of an engineered PspA recombinant antigen against *Streptococcus pneumoniae* . Biotechnol Rep (2019) 24:1–7. doi: 10.1016/j.btre.2019.e00385 PMC6864353 31763198

[33] Afshari E Amini-Bayat Z Hosseinkhani S Bakhtiari N. Cloning, expression and purification of pseudomonas putida ATCC12633 creatinase. Avicenna J Med Biotechnol (2017) 9:169–75. PMC5650733 29090065

[34] Ahmadi K Pouladfar G Kalani M Faezi S Pourmand MR Hasanzadeh S. Epitope-based immunoinformatics study of a novel Hla-MntC-SACOL0723 fusion protein from *Staphylococcus aureus*: Induction of multi-pattern immune responses. Mol Immunol (2019) 114:88–99. doi: 10.1016/j.molimm.2019.05.016 31351414

[35] Kielkopf CL Bauer W Urbatsch IL. Bradford assay for determining protein concentration. Cold Spring Harbor Protoc (2020) 2020:102269. doi: 10.1101/pdb.prot102269 32238597

[36] Malekan M Siadat SD Aghasadeghi M Shahrokhi N Afrough P Behrouzi A. Evaluation of protective immunity responses against pneumococcal PhtD and its C-terminal in combination with outer-membrane vesicles as adjuvants. J Med Microbiol (2020) 69:465–77. doi: 10.1099/jmm.0.001103 32100705

[37] Cao J Chen D Xu W Chen T Xu S Luo J. Enhanced protection against pneumococcal infection elicited by immunization with the combination of PspA, PspC, and ClpP. Vaccine (2007) 25:4996–5005. doi: 10.1016/j.vaccine.2007.04.069 17524530

[38] Ogunniyi AD Grabowicz M Briles DE Cook J Paton JC. Development of a vaccine against invasive pneumococcal disease based on combinations of virulence proteins of Streptococcus pneumoniae. Infection Immun (2007) 75:350–7. doi: 10.1128/IAI.01103-06 PMC1828427 17088353

[39] Denoël P Philipp MT Doyle L Martin D Carletti G Poolman JT. A protein-based pneumococcal vaccine protects rhesus macaques from pneumonia after experimental infection with Streptococcus pneumoniae. Vaccine (2011) 29:5495–501. doi: 10.1016/j.vaccine.2011.05.051 PMC5061031 21624422

[40] Afrough P Bouzari S Mousavi SF Karam MRA Vaziri F Fateh A. Evaluation of immunological responses to recombinant Porin A protein (rPoA) from native strains of *Neisseria meningitidis* serogroups A and B using OMV as an adjuvant in BALB/c mice. Microbial Pathogenesis (2017) 112:209–14. doi: 10.1016/j.micpath.2017.09.038 28942175

[41] Tada R Suzuki H Takahashi S Negishi Y Kiyono H Kunisawa J. Nasal vaccination with pneumococcal surface protein A in combination with cationic liposomes consisting of DOTAP and DC-chol confers antigen-mediated protective immunity against Streptococcus pneumoniae infections in mice. Int Immunopharmacol (2018) 61:385–93. doi: 10.1016/j.intimp.2018.06.027 29945026

[42] Cao J Zhang X Gong Y Zhang Y Cui Y Lai X. Protection against pneumococcal infection elicited by immunization with multiple pneumococcal heat shock proteins. Vaccine (2013) 31:3564–71. doi: 10.1016/j.vaccine.2013.05.061 23727004

[43] Wolf JJ. Special Considerations for the Nonclinical Safety Assessment of Vaccines. In: Nonclinical Development of Novel Biologics, Biosimilars, Vaccines and Specialty Biologics. Academic Press: Elsevier (2013). p. 243–255. doi: 10.1016/B978-0-12-394810-6.00010-1

[44] Siadat SD Naddaf SR Zangeneh M Moshiri A Sadat SM Ardestani MS. Outer membrane vesicle of Neisseria meningitidis serogroup B as an adjuvant in immunization of rabbit against Neisseria meningitidis serogroup A. Afr J Microbiol Res (2011) 5:3090–5. doi: 10.5897/AJMR11.361

[45] Palumbo E Fiaschi L Brunelli B Marchi S Savino S Pizza M. Antigen identification starting from the genome: a “Reverse Vaccinology” approach applied to MenB. In: Neisseria meningitidis. Humana, Totowa, NJ: Springer: Academic Press (2012). p. 361–403. doi: 10.1007/978-1-61779-346-2_21 21993656

[46] André GO Assoni L Rodriguez D Leite LCC Dos Santos TEP Ferraz LFC. Immunization with PhtD truncated fragments reduces nasopharyngeal colonization by Streptococcus pneumoniae. Vaccine (2020) 38:4146–53. doi: 10.1016/j.vaccine.2020.04.050 32362528

[47] Norolahi F Siadat SD Malekan M Mousavi SH Janani A Mousavi SF. Relationship between prevalence of pneumococcal serotypes and their neuraminidases in carriers, predictive facts? Arch Pediatr Infect Dis (2020) 8:e14100. doi: 10.5812/pedinfect.14100

[48] Zhou M Wang Z Zhang L Kudinha T An H Qian C. Serotype distribution, antimicrobial susceptibility, multilocus sequencing type and virulence of invasive Streptococcus pneumoniae in China: a six-year multicenter study. Front Microbiol (2021) 12. doi: 10.3389/fmicb.2021.798750 PMC8793633 35095809

[49] Lagousi T Basdeki P De Jonge MI Spoulou V. Understanding host immune responses to pneumococcal proteins in the upper respiratory tract to develop serotype-independent pneumococcal vaccines. Expert Rev Vaccines (2020) 19:959–72. doi: 10.1080/14760584.2020.1843433 33107359

[50] Afshari E Ahangari Cohan R Sotoodehnejadnematalahi F. In-silico analysis of pneumococcal heat-shock protein (DnaJ) to predict novel multi-epitope vaccine candidates. Vaccine Res (2021) 8:65–87. doi: 10.52547/vacres.8.2.65

[51] Brooks LR Mias GI. *Streptococcus pneumoniae*’s virulence and host immunity: aging, diagnostics, and prevention. Front Immunol (2018) 9:1366. doi: 10.3389/fimmu.2018.01366 29988379 PMC6023974

[52] Scelfo C Menzella F Fontana M Ghidoni G Galeone C Facciolongo NC. Pneumonia and invasive pneumococcal diseases: the role of pneumococcal conjugate vaccine in the era of multi-drug resistance. Vaccines (2021) 9:420. doi: 10.3390/vaccines9050420 33922273 PMC8145843

[53] Chiavolini D Pozzi G Ricci S. Animal models of *Streptococcus pneumoniae* disease. Clin Microbiol Rev (2008) 21:666–85. doi: 10.1128/CMR.00012-08 PMC2570153 18854486

[54] Briles DE Nahm M Schroer K Davie J Baker P Kearney J. Antiphosphocholine antibodies found in normal mouse serum are protective against intravenous infection with type 3 streptococcus pneumoniae. J Exp Med (1981) 153:694–705. doi: 10.1084/jem.153.3.694 7252411 PMC2186108

[55] Briles DE Hollingshead SK Jonsdottir I. Animal models of invasive pneumococcal disease. In: Pneumococcal Vaccines: Impact Conjugate Vaccines (2008). p. 47–58. doi: 10.1128/9781555815820.ch4

[56] Ogunniyi AD Folland RL Briles DE Hollingshead SK Paton JC. Immunization of mice with combinations of pneumococcal virulence proteins elicits enhanced protection against challenge with Streptococcus pneumoniae. Infection Immun (2000) 68:3028–33. doi: 10.1128/IAI.68.5.3028-3033.2000 PMC97524 10769009

[57] Wu K Zhang X Shi J Li N Li D Luo M. Immunization with a combination of three pneumococcal proteins confers additive and broad protection against *Streptococcus pneumoniae* infections in mice. Infection Immun (2010) 78:1276–83. doi: 10.1128/IAI.00473-09 PMC2825939 20038538

[58] Morais V Texeira E Suarez N. Next-generation whole-cell pneumococcal vaccine. Vaccines (2019) 7:151. doi: 10.3390/vaccines7040151 31623286 PMC6963273

[59] Shi S Zhu H Xia X Liang Z Ma X Sun B. Vaccine adjuvants: Understanding the structure and mechanism of adjuvanticity. Vaccine (2019) 37:3167–78. doi: 10.1016/j.vaccine.2019.04.055 31047671

[60] Malekan M Siadat SD Aghasadeghi M Shahrokhi N Eybpoosh S Afshari E. Assessment of phtD C-terminal immunogenicity by opsonophagocytosis assay (OPA) with OMVs as adjuvants. Vaccine Res (2019) 6:37–41. doi: 10.29252/vacres.6.2.37

[61] Kothari N Kothari S Choi YJ Dey A Briles DE Rhee DK. A bivalent conjugate vaccine containing PspA families 1 and 2 has the potential to protect against a wide range of Streptococcus pneumoniae strains and Salmonella Typhi. Vaccine (2015) 33:783–8. doi: 10.1016/j.vaccine.2014.12.032 25545593

[62] Wang Y Xia L Wang G Lu H Wang H Luo S. Subcutaneous immunization with the fusion protein ΔA146Ply-SP0148 confers protection against Streptococcus pneumoniae infection. Microbial Pathogenesis (2022) 162:105325. doi: 10.1016/j.micpath.2021.105325 34848296

[63] Su Y Li D Xing Y Wang H Wang J Yuan J. Subcutaneous immunization with fusion protein DnaJ-ΔA146Ply without additional adjuvants induces both humoral and cellular immunity against Pneumococcal infection partially depending on TLR4. Front Immunol (2017) 8:686. doi: 10.3389/fimmu.2017.00686 28659923 PMC5466963

[64] Wu Y Cui J Zhang X Gao S Ma F Yao H. Pneumococcal DnaJ modulates dendritic cell-mediated Th1 and Th17 immune responses through Toll-like receptor 4 signaling pathway. Immunobiology (2017) 222:384–93. doi: 10.1016/j.imbio.2016.08.013 27594384

[65] Heeb LE Egholm C Boyman O. Evolution and function of interleukin-4 receptor signaling in adaptive immunity and neutrophils. Genes Immun (2020) 21:143–9. doi: 10.1038/s41435-020-0095-7 PMC7274943 32139893

[66] Ike K Uchida Y Nakamura T Imai S. Induction of interferon-gamma (IFN-γ) and T helper 1 (Th1) immune response by bitter gourd extract. J Veterinary Med Sci (2005) 67:521–4. doi: 10.1292/jvms.67.521 15942138

[67] Kak G Raza M Tiwari BK. Interferon-gamma (IFN-γ): Exploring its implications in infectious diseases. Biomolecular Concepts (2018) 9:64–79. doi: 10.1515/bmc-2018-0007 29856726

[68] Weiser JN Ferreira DM Paton JC. *Streptococcus pneumoniae*: transmission, colonization and invasion. Nat Rev Microbiol (2018) 16:355–67. doi: 10.1038/s41579-018-0001-8 PMC5949087 29599457

[69] Shaper M Hollingshead SK Benjamin WH Jr Briles DE. PspA protects *Streptococcus pneumoniae* from killing by apolactoferrin, and antibody to PspA enhances killing of pneumococci by apolactoferrin. Infection Immun (2004) 72:5031–40. doi: 10.1128/IAI.72.9.5031-5040.2004 PMC517438 15321996

[70] Kell DB Heyden EL Pretorius E. The biology of lactoferrin, an iron-binding protein that can help defend against viruses and bacteria. Front Immunol (2020) 11:1221. doi: 10.3389/fimmu.2020.01221 32574271 PMC7271924

[71] Senkovich O Cook WJ Mirza S Hollingshead SK Protasevich II Briles DE. Structure of a complex of human lactoferrin N-lobe with pneumococcal surface protein a provides insight into microbial defense mechanism. J Mol Biol (2007) 370:701–13. doi: 10.1016/j.jmb.2007.04.075 PMC5356469 17543335

[72] Xu Q Surendran N Verhoeven D Klapa J Ochs M Pichichero ME. Trivalent pneumococcal protein recombinant vaccine protects against lethal *Streptococcus pneumoniae* pneumonia and correlates with phagocytosis by neutrophils during early pathogenesis. Vaccine (2015) 33:993–1000. doi: 10.1016/j.vaccine.2015.01.014 25597944

